# Supplementary material for: Transcriptome of the coralline alga Calliarthron tuberculosum (Corallinales, Rhodophyta) reveals convergent evolution of a partial lignin biosynthesis pathway
Source: PLoS One. 2022 Jul 14;17(7):e0266892. doi: 10.1371/journal.pone.0266892 (PMC9282553; doi:10.1371/journal.pone.0266892)
Supplement: S1 Appendix — Genome reassembly and removal of contamination; analysis of completeness for genomic and transcriptomic datasets; E-value thresholds used in gene candidate identification; accession numbers for all sequences used; identification of cytochrome P450s sequences (C3H, C4H, and F5H), CCoAOMT, and CSE; and the benefits and limitations of using KEGG mapping for gene annotation of Calliarthron sequences. (DOCX) [file pone.0266892.s001.docx]

**Appendix S1: Supplemental Methods and Results**

**Transcriptome of the coralline alga *Calliarthron tuberculosum* (Corallinales, Rhodophyta) reveals convergent evolution of a partial lignin biosynthesis pathway**

Table of contents …...……………………………………………………………..………………1

Genome reassembly and removal of contamination ……………………….…………..………....2

Completeness of genome and transcriptome datasets ……………....…………………….………2

E-value cut offs for HMMR searches ……….………………………..………………………….3

Accession numbers for *4CL* analysis (Fig 2) ……………………..…………………...………….4

Accession numbers for *CCR* analysis (Fig 3) …………….………………………...…………….6

Accession numbers for *CAD* analysis (Fig 4) ……………..……………………………………...7

Identification of *Cytochrome P450s sequences C3H, C4H, and F5H* ……………...……………8

Accession numbers for *Cytochrome P450s (C3H, C4H, F5H)* analysis (Fig S2) .……………...10

Identification of *CCoAOMT* ………………………………………………………..…………...12

Accession numbers for *CCoAOMT* analysis (Fig S3)…………………………...………………13

#### Identification of CSE ……………………………………………...……………………………..14

Accession numbers for *CSE* analysis (Fig S4) ………………………………………………….15

Benefits and limitations of extracting KEGG metabolic pathways …………….……………….17

References ……………………………………………………………………………………….18

**Genome reassembly and removal of contamination**

Of the assembled scaffolds from *Calliarthron tuberculosum*, 46 were identified as putative bacterial or viral sequences and removed. The genome assembly without scaffolding from the transcriptome consists of 48,101 scaffolds (N50 statistic length 2.99Kb). Genome assembly using the transcriptome as a scaffold improved the assembly slightly to 49,536 scaffolds (N50 statistic length 2.858 Kb). To further explore the genomic reassembly for contamination, the genomic G+C content was assessed separately for scaffolds with transcriptome support and without transcriptome support. The 21,672 scaffolds (45.0% of 48,147) supported by transcriptome evidence have a higher G+C content (mean GC 45%) compared to the 26,429 scaffolds that do not have transcriptome support (mean GC 39%) (Fig S1). In total there are 26,429 genomic scaffolds (21,672 contigs, N50 3.867 Kb, total assembled bases 64.152 Mbp).

**Completeness of genomic and transcriptomic datasets**

Genome and transcriptome data completeness was assessed by the recovery of core conserved eukaryotic genes using the CEGMA [1] and BUSCO [2] datasets. The 21,672 genome scaffolds (with transcriptome support) and the combined transcriptome are highly complete (CEGMA: >93% completeness, BUSCO: >74% completeness) (Fig S1). BUSCO is stricter than CEGMA for non-model organisms. The distribution of genomic scaffolds from *C. tuberculosum* shared with genomic sequences from the red alga *Pyropia yezoensis* was assessed with the remaining sequences categorized into eukaryotic or bacterial origins based on sequence similarity (Table S1). The majority of genomic scaffolds from *C. tuberculosum* have shared sequence similarity with *P. yezoensis* (2,125 scaffolds). The *C. tuberculosum* dataset contained 180 eukaryotic scaffolds and 847 bacterial scaffolds not shared with the *P. yezoensis* dataset (Table S1).

**E-value cut offs for HMMER searches**

| Gene candidates | e-value cut off |
| --- | --- |
| 4CL | 1e-45 |
| CCR | 1e-25 |
| CAD | 1e-13 |
| CYP450 | 1e-20 |
| CCoAOMT | 1e-20 |
| CSE | 1e-6 |
| PAL/TAL/PTAL | 1e-7 |

####

#### Accession numbers for 4CL analysis (Fig 2)

Fig 2A:

AAF37732 *Lolium perenne* Lp4CL1, AAF37733 *Lolium perenne* Lp4CL2, ACD02135 *Panicum virgatum* Pv4CL1, AAS67644 *Zea mays* Zm4CL1, XP_002969881 *Selaginella moellendorffii* Sm4CL1, AAL98709 *Glycine max* Gm4CL1, ACF35279 *Pinus radiate* Pr4CL1, NP_175579 *Arabidopsis thaliana* At4CL1, AAC24503 *Populus tremuloides* Pt4CL1, O24145 *Nicotiana tabacum* Nt4CL1, O24146 *Nicotiana tabacum* Nt4CL2.

Fig 2B:

AAC24503, AAC37253, AAC37254, AAF37732, AAF37733, AAL98709, AAS67644, ABV60447, ABV60448, ABV60449, ACD02135, ACF35279, AMT98406, AT1G20480, AT1G20490, AT1G20500, AT1G20510, AT1G20560, AT1G21530, AT1G21540, AT1G23160, AT1G28130, AT1G30520, AT1G48660, AT1G48670, AT1G49430, AT1G51680, AT1G59500, AT1G62940, AT1G64400, AT1G65880, AT1G65890, AT1G66120, AT1G68270, AT1G75960, AT1G76290, AT1G77240, AT1G77590, AT2G04350, AT2G14960, AT2G17650, AT2G23170, AT2G46370, AT2G47240, AT2G47750, AT3G05970, AT3G16170, AT3G16910, AT3G21230, AT3G21240, AT3G48990, AT4G03400, AT4G11030, AT4G14070, AT4G19010, AT4G23850, AT4G27260, AT4G37390, AT5G05160, AT5G13320, AT5G13350, AT5G13360, AT5G13370, AT5G13380, AT5G16370, AT5G23050, AT5G27600, AT5G36880, AT5G38120, AT5G51470, AT5G54510, AT5G63380, BAK12046, c139317_g1_i1_4, c139587_g1_i1_5, c141558_g4_i1_3, c141558_g4_i2_3, c141618_g1_i1_3, c141893_g1_i1_3, c142036_g1_i1_1, c142563_g1_i1_2, c145839_g1_i1_1, c95137_g1_i1_5, CAA59281, CAA9363949, CAB4104811, CBJ27533, CBJ28329, CDF32396, CDF41291, EEC50893, EED93473, EFN52634, EFN53018, EFN55808, EFX65682, EIE20280, EKU22239, EKX33608, EME31572, EME32155, EME32164, EME32820, EME32859, HAO13495, KAA8490963, KAA8493489, KAA8494473, KAA8494587, KAA8497056, KAA8497564, KAF0916871, KAF6004312, KAF7101945, KAF7101946, KAF8061938, KAG0584171, KAG0588707, KAG0623632, KAG0627631, KAG2439883, KAG2716051, KAG2716056, KXZ55413, MBF1991334, MBI2191916, MBL7850533, NBO48550, NWG38034, O24145, O24146, OEU13519, OQR96481, OSX69535, OSX70295, OSX74244, OSX79543, PNR28027, PNW73635, PXF40762, PXF41346, PXF44881, PXF47267, PXF47592, PXF47672, PXF48392, QGN03526, RMG86589, SOD04037, VAI88204, WP_013961719, WP_016869319, WP_026951742, WP_079681472, WP_102171029, WP_102220786, WP_135073957, WP_207947089, XP_001747327, XP_001747423, XP_002739084, XP_002948463, XP_002968990, XP_002969881, XP_002969881, XP_002990653, XP_005535676, XP_005705549, XP_005707610, XP_005712755, XP_005714920, XP_005715308, XP_005716938, XP_005717654, XP_005718027, XP_005771927, XP_006415459, XP_012067417, XP_012067419, XP_018827690, XP_020916102, XP_020916103, XP_021313453, XP_021627094, XP_021627095, XP_022885537, XP_023732870, XP_024364789, XP_028672029, XP_031486777, XP_033858027, XP_034768253, XP_034768254, XP_039622562

#### Accession numbers for CCR analysis (Fig 3)

Fig 3A:

AAG46037 *Arabidopsis thaliana* AtCCR1, CAA56103 *Eucalyptus gunnii* EgCCR, AAN71760 *Hordeum vulgare* HvCCR, AAG09817 *Lolium perenne* LpCCR, AAL47684 *Pinus taeda* PtCCR, XP_002303845 *Populus trichocarpa* PtCCR, AAN71761 *Solanum tuberosum* StCCR, ABE01883 *Triticum aestivum* TaCCR1, AAX08107 *Triticum aestivum* TaCCR2.

Fig 3B:

AT1G09480, AT1G09490, AT1G09500, AT1G09510, AT1G15950, AT1G51410, AT1G66800, AT1G76470, AT1G80820, AT2G33590, AT2G33600, AT2G45400, AT4G27250, AT4G35420, AT5G19440, AT5G42800, BAH56929, c118951_g1_i1_2, c124623_g1_i1_2, c95473_g1_i1_5, CAA69253, CCA15100, EDK31897, EEC43073, EED93941, EFJ06723, EFJ22121, EFJ32728, EFJ35085, EFN54625, EGD83125, EIE25032, EIE25033, EKU22444, EKX50916, KAA3644909, KAF2205997, KAF2268117, KAF2793960, KAF9688883, KZK75114, MBL8632307, MBL9003083, NCC73432, OAL49492, OEU20249, OEU20938, OSS53899, PIA48431, PIA48432, PIA48433, PSN66757, PXF42666, TLU56187, TNC82432, WP_006366457, WP_085659952, XP_001020008, XP_001027418, XP_001028134, XP_001637357, XP_001702360, XP_002117601, XP_002675494, XP_002908946, XP_002950118, XP_002956636, XP_003386068, XP_003834537, XP_004349571, XP_004349572, XP_005713248, XP_005766535, XP_005846728, XP_005851647, XP_012702439, XP_022934191, XP_022982785, XP_023526588, XP_023526659, XP_626064, XP_626064

#### Accession numbers for CAD analysis (Fig 4)

Fig 4A:

P06525 *Arabidopsis thaliana* ADH1, AT4G34230 *Arabidopsis thaliana* AtCAD5, AT4G37990 *Arabidopsis thaliana* AtCAD8, AT4G39330 *Arabidopsis thaliana* AtCAD9, ADP68542 *Pinus armandii* PaCAD.

Fig 4B:

AAF73254, AAK26851, AAK26852, ABD46589, ABK21418, ABK26019, ABK27011, ABR16979, ADP68542, ADP68549, ADP68550, AOR07019, AT1G72680, AT2G21730, AT2G21890, AT3G19450, AT4G34230, AT4G37970, AT4G37980, AT4G37990, AT4G39330, c121452_g1_i1_5, c121452_g1_i2_5, c137498_g1_i1_3, c137515_g1_i1_2, c145607_g1_i1_5, CAD7623026, CAE7171048, CAE7264721, CAE7476856, CAE7562051, CAE7658906, CAE7947340, CBY84994, CCA26192, CCO14774, CCO17147, CCO19759, CDF34840, EAR92957, EEC44695, EEC46746, EED94449, EED96219, EED96421, EFN56047, EFN56723, EGZ11446, EIE27722, ELU06025, EME26563, EME27044, EME27893, OEU10098, OEU20286, OKL43300, OLQ07033, P06525, P25377, P27250, P75691, PXF42146, PXF46026, PXF46046, PXF48590, PXF48875, Q04894, RMX48738, WP_064640725, WP_138237030, WP_177185849, WP_192269644, XP_001416318, XP_001421868, XP_001422100, XP_001441600, XP_001624910, XP_001693170, XP_002139197, XP_002501541, XP_002902902, XP_002952441, XP_002957700, XP_003057538, XP_003061453, XP_005711469, XP_005713108, XP_005713109, XP_005773924, XP_005792427, XP_013419139, XP_020895616, XP_021373496, XP_022788711, XP_027040389, XP_028413386, XP_035232319, XP_626204, XP_667966

#### Identification of Cytochrome P450s sequences C3H, C4H, and F5H

Coumarate 3-hydroxylase (C3H), cinnamate 4‐hydroxylase (C4H), and ferulate 5-hydroxylase (F5H) are cytochrome P450 (CYP) monooxygenases that catalyze hydroxylation reactions required for horizontal movement through the monolignol pathway to produce monolignols that correspond to the H, G and S lignin subunits (for a review see [4]). In addition to monolignol biosynthesis, these enzymes branch the phenylpropanoid biosynthesis pathway for the production of other flavanoids (reviewed in [5]). We identified six sequences as candidate C3H, C4H, or F5H encoding transcripts, based on HMMER searches but not based on KEGG analysis. These candidate sequences were not uniquely identified as either C3H, C4H, or F5H, but overlap in their identification. This overlap is not surprising as C3H, C4H, and F5H are all closely related CYP sequences together in the CYP71 clan that are thought to have diverged in land plants (for a review of CYPs see [6]). A query of these sequences against the *Arabidopsis thaliana* proteome returned other sequences within the CYP71 clade that C3H, C4H, and F5H belong to as well, as the CYP85 clan is involved in terpenoid plant hormone metabolism (Table S2). For CYP monooxygenase function, the K loop is important in stabilizing the tertiary structure while the PXRX motif and I helix are involved in oxygen binding and activation. These domains show high conservation in key residues (identity similarity [IS] >90% Fig S2A). The heme binding domain also shows high conservation in key residues (IS >80% Fig S2A).

In the CYP gene tree analysis, *C. tuberculosum* CYP sequence candidates fall within three general groupings. One of the sequence candidates groups within the CYP97 clade with high confidence (ultrafastbootstrap [BS] = 100%, Fig S2A and S2B). This *C. tuberculosum* sequence is likely involved in carotenoid biosynthesis and not monolignol biosynthesis based on previous functional annotation of land plant sequences in this clade [7]. The second group of *C. tuberculosum* sequences forms a clade with other red algal sequences (BS = 100%) (Fig S2B) and likely represents a novel family or clade of CYPs that have evolved in the red algal lineage. The third group includes *C. tuberculosum* and other red algal sequences and has been annotated as an algal CYP71 clade based on sequence annotation but does not have support as the sister clade to the plant CYP71s (BS = 77%). This group would be the best candidate for a *Calliarthron* C3H, C4H, or F5H, but requires further functional evidence. The CYP71 clan that contains the true C3H, C4H, and F5H from *A. thaliana* forms a separate clade and has likely independently evolved within the rapidly diversified CYP71 clade in land plants.

**Accession numbers for *Cytochrome P450s (C3H, C4H, F5H)* analysis (Fig S2)**

Fig S2A:

NP_850337.1 *Arabidopsis thaliana* AtC3H, ABV80343.1 *Selaginella moellendorffii* SmF5H, CAP08841.1 *Arabidopsis thaliana* AtC4H, Q42600.1 *Arabidopsis thaliana* AtF5H.

Fig S2B:

AB005237, AB005248, AB007645, AB007647, AB007648, AB007649, AB009048, AB010073, AB010077, AB010692, AB010697, AB010697, AB011485, AB016885, AB016889, AB016893, AB017064, AB017064, AB017064, AB017064, AB017065, AB018112, AB018112, AB019226, AB019226, AB019233, AB019233, AB020744, AB022210, AB022210, AB023038, AB023038, AB023038, AB023038, AB023038, AB023038, AB023038, AB023038, AB023038, AB024024, AB024038, AB024038, AB024038, AB024038, AB024038, AB024038, AB024038, AB024038, AB024038, AB024038, AB024038, AB024038, AB024038, AB024038, AB025602, AB025602, AB025623, AB026647, AB026661, AC000098, AC000103, AC001229, AC001304, AC002304, AC002329, AC002340, AC002340, AC002388, AC002391, AC002391, AC002391, AC002409, AC002561, AC003105, AC003680, AC003680, AC003680, AC003680, AC003680, AC004077, AC004077, AC004136, AC004146, AC004411, AC004411, AC004484, AC004512, AC004665, AC005272, AC005315, AC005405, AC005623, AC005623, AC005700, AC005727, AC005819, AC005824, AC005897, AC005964, AC005964, AC005967, AC006193, AC006193, AC006193, AC006193, AC006259, AC006259, AC006341, AC006341, AC006592, AC006592, AC006931, AC007018, AC007019, AC007202, AC007260, AC007296, AC007296, AC007296, AC007296, AC007323, AC007357, AC007357, AC007357, AC007357, AC007357, AC007357, AC007357, AC007519, AC007519, AC007651, AC008051, AC008051, AC008051, AC009895, AC010155, AC010164, AC010164, AC010795, AC010797, AC011560, AC011560, AC011622, AC011765, AC011765, AC012561, AC012561, AC012561, AC013258, AC016662, AC023279, AC023628, AC024226, AC024609, AC025417, AC026480, AC027656, AC061957, AC073178, AC074025, AC079041, AC079733, AF069716, AF069716, AF069716, AF069716, AF195115, AHA51695, AL021635, AL021635, AL021636, AL021636, AL021636, AL021687, AL021811, AL022141, AL022224, AL022224, AL031369, AL035528, AL035601, AL035601, AL035601, AL035601, AL035601, AL035602, AL035708, AL049608, AL049608, AL049659, AL049659, AL049659, AL049659, AL049659, AL049659, AL078620, AL078620, AL078620, AL078620, AL080283, AL080318, AL080318, AL080318, AL080318, AL132958, AL132958, AL132958, AL132958, AL132958, AL132969, AL132979, AL133315, AL133421, AL138642, AL162875, AL162972, AL162973, AL163817, AL163972, AL353865, AL353992, AL353995, AL353995, AL357612, AL358732, AL358732, ANT70528, AP000383, AP000383, AP000383, AP000383, AP000383, AP000383, AP000383, AP000383, AP000383, AP000383, AP000414, AP000419, AP001298, AP001298, AP001298, AP001298, AP001298, AP001304, AP001304, AP001304, AP001304, AP001304, AP001307, AP001314, AP002033, AP002057, AP002060, c121208_g1_i1_4, c121947_g1_i1_5, c128384_g1_i1_5, c128448_g1_i1_3, c134457_g1_i1_3, c55657_g1_i1_3 , CYP720A1, CYP84A4, EWM30289, KAA8490514, KAA8492888, KAA8494777, KAF6001237, KAG5183231, OCC28276, OSX72062, OSX75164, OSX75165, PXF41140, PXF46519, QJT93736, TDX02322, U93215, KAA8494777_, KAF6001237_, KAG5183231_, OCC28276_, OSX72062_, OSX75164_, OSX75165_, PXF41140_, PXF46519_, QJT93736_, TDX02322_, XP_3074608, XP_5535456, XP_5703131, XP_5703132, Z97337, Z97338, Z97338, Z97338, Z97338, Z97338, Z97338, Z97338, Z97338, Z97338, Z97338, Z97339, Z99707, Z99707, Z99707, Z99707, Z99708

#### Identification of CCoAOMT

Caffeoyl CoA O-methyl-transferase (CCoAOMT) converts Caffeoyl-CoA into Feruloyl-CoA [10] for one route in G and S lignin subunit production. We identified three transcripts as candidate CCoAOMT-encoding sequences: two based on KEGG analysis and one additional sequence based on HMMER searches. A query of these sequences against the *Arabidopsis* proteome returned CCoAOMT and putative CCoAOMT sequences (Table S2). Sequence conservation at substrate recognition sites [11, 12] are completely conserved at some sites (IS = 100%) and have little to no conservation at other sites (IS <30%) (black triangles Fig S3A). Divalent metal binding sites also appear to be conserved (IS >80%) (grey triangles Fig S3A) with the D203N204 critical for Ca^+2^ coordination. Residues thought to be important for catalytic activity (black square) and electrostatic interactions with the caffeoyl-CoA substrate (grey square) are completely conserved (IS = 100%) (Fig S3A).

In CCoAOMT gene tree analysis, the *C. tuberculosum* sequences group within two general clades (Fig S3B). One sequence is sister to the land plant CCoAOMT clade with low support (BS = 89%) and a likely CCoAOMT candidate. Another two *C. tuberculosum* sequences are found within the algal OMT group (BS = 90%). The land plant CCoAOMTs are sister to and have a closer relationship with the algal OMT group (BS = 100%) than other plant OMTs such as the flavanoid OMT. This suggests that the ancestor of the algal OMT, including *C. tuberculosum* sequences, and plant CCoAOMT sequences may have emerged prior to the divergence of these lineages.

**Accession numbers for *CCoAOMT* analysis (Fig S3)**

Fig S3A:

Q40313 *Medicago sativa* MsCCOAOMT, Q43237 *Vitis vinifera* VvCCOAOMT, O65862 *Populus trichocarpa* PtCCoAOMT1, P28034 *Petroselinum crispum* PtCCoAOMT, Q9ZTT5 *Pinus taeda* PtCCoAOMT, O24144 *Nicotiana tabacum* NtCCoAOMT, Q41720 *Zinnia violacea* ZvCCoAOMT, Q43161 *Stellaria longipes* SlCCoAOMT, Q6YI95 *Mesembryanthemum crystallinum* McCCoAOMT.

Fig S3B:

ALX18653, ABF61767, c140748_g1_i1_5, c142188_g1_i1_2, c147617_g1_i1_1, CEM30980, EJK69238, EWM25750, GAX12418, GAX24973, KAA8497666, O04385, O22381, O24144, O24305, O24529, O65862, OSX70340, P28002, P28034, P45986, P93324, PXF42168, PXF46781, PXF47808, Q00763, Q06509, Q38J50, Q39522, Q40313, Q41720, Q42653, Q42654, Q42949, Q43096, Q43161, Q43237, Q43239, Q43771, Q6L8K4, Q6T1F5, Q6T1F6, Q6VMV8, Q6VMV9, Q6VMW0, Q6VMW1, Q6VMW2, Q6WUC1, Q6WUC2, Q6YI95, Q84KK4, Q84KK5, Q84KK6, Q84XW5, Q8GSN1, Q8H9A8, Q8L5K7, Q8L5K8, Q8W013, Q93WU2, Q93WU3, Q96565, Q9AR07, Q9FK25, Q9FYZ9, Q9LEL5, Q9LEL6, Q9SPV4, Q9SWR0, Q9SYR8, Q9SYR9, Q9XE90, Q9XE91, Q9ZTT5, TFJ85214, TRY76935, VEU41255, XP_001632538, XP_002185078, XP_005712596, XP_005712784, XP_005715311, XP_005718383, XP_020912350, XP_029206255, XP_031553077, XP_031553078

#### Identification of CSE

Caffeoyl shikimate esterase (CSE) removes the shikimate decoration and converts caffeoyl shikimate into caffeic acid, shifting monolignol production towards G and S lignin subunits [14, 15]. We identified eight transcripts as candidate CSE-encoding transcripts based on HMMER searches, but the KEGG analysis did not identify any CSE candidates. A query of these sequences against the *Arabidopsis* proteome returned CSE and other proteins within the α/β‐hydrolase superfamily that CSE belongs to (Table S2). The *C. tuberculosum* sequences have large insertions in domains such as the first GXSXG conserved motif (Fig S4A).

In gene tree analysis of the *C. tuberculosum* sequences and sequences from *A. thaliana,* the α/β‐hydrolase superfamily, to which CSE belongs, two *C. tuberculosum* sequences group with other red algal sequences and the *A. thaliana* monoacylglycerol lipase (MAGL) clade with low support (BS = 80; Fig S4B). These two identified *C. tuberculosum* sequences are our best CSE homolog candidates, as CSE belongs to the MAGL subfamily (Fig S4B). Additional *C. tuberculosum* sequences are found throughout the tree with very low support in the backbone, preventing functional predictions of these candidates.

**Accession numbers for *CSE* analysis (Fig S4)**

Fig S4A:

XP_002298118.1 Populus trichocarpa, AAP42742.1 Arabidopsis thaliana, c134094_g1_i1_1, c77395_g1_i2_6, c77395_g1_i1_6, c46429_g1_i1_3, c141919_g1_i1_2, c139612_g1_i1_2, c141828_g1_i2_1, c141828_g1_i3_3

Fig S4B:

AB00639, AT1G11090, AT1G18360, AT1G52760, AT1G73480, AT1G77420, AT2G39400, AT2G39410, AT2G39420, AT2G47630, AT3G55180, AT3G55190, AT3G62860, AT5G11650, AT5G14980, AT5G16120, AT5G19290, c134094_g1_i1_1, c139612_g1_i1_2, c141828_g1_i2_1, c141828_g1_i3_3, c141919_g1_i1_2, c46429_g1_i1_3, c77395_g1_i1_6, c77395_g1_i2_6, CBJ28544, CBJ28545, CDW72798, EOY26593, F4HXL0, F4I0K9, F4IE65, F4IMK2, F4IMK4, F4JFU8, F4JRA6, GAQ79936, GBG27369, HGX14611, KAA8491713, KAA8495708, KAA8528800, KAF6003208, KAG2495972, KAG2500907, KAG2692888, KXZ48785, KXZ55885, KYR00280, MAY36621, MBC13192, MBN2296663, MBN2580056, MBO9367125, MBQ0758220, NEQ98214, NLB71918, O04084, O22527, O22975, O22977, O23171, O23364, O23512, O23522, O49523, O64640, O64641, O64810, O64811, O80472, O80474, O80475, O80476, O80477, O81009, O82229, O82274, OFW63598, OSX75617, P32826, P93732, PXF40937, PXF43372, PXF43429, PXF43616, PXF46973, PXF47053, Q0WPR4, Q0WRX3, Q15KI9, Q1PET6, Q1PF08, Q1PF50, Q2V465, Q3EBR6, Q4F883, Q4PSY2, Q4VCM1, Q56WF8, Q67Y83, Q67ZU1, Q680C0, Q6DBP4, Q700D5, Q71DJ5, Q71N54, Q7Y220, Q84LM4, Q84W27, Q84WF0, Q84WK4, Q8GYK2, Q8H780, Q8L7B2, Q8L9Y0, Q8LAS8, Q8LED9, Q8LFX7, Q8RUW5, Q8RWJ6, Q8RXP6, Q8S8K6, Q8S8P0, Q8S8S9, Q8S9K8, Q8VY01, Q8VYP9, Q8VZF3, Q8VZU3, Q8W5R2, Q93V61, Q93Y09, Q940G6, Q940H7, Q940L4, Q941F1, Q948R1, Q949Q7, Q94AC1, Q94AS5, Q9ASU8, Q9C7D2, Q9C7D3, Q9C7D4, Q9C7D6, Q9C7Z9, Q9C8J6, Q9CAU0, Q9CAU1, Q9CAU2, Q9CAU3, Q9CAU4, Q9FFB0, Q9FFB2, Q9FFZ1, Q9FG13, Q9FH05, Q9FH06, Q9FJ24, Q9FJT7, Q9FN79, Q9FNA9, Q9FVW3, Q9FW03, Q9FX92, Q9FX93, Q9FX94, Q9FYC7, Q9FZ33, Q9FZI8, Q9LEY1, Q9LFR7, Q9LFT6, Q9LK21, Q9LMA7, Q9LNC2, Q9LSM9, Q9LSV8, Q9LT10, Q9LVB8, Q9LVL9, Q9LW26, Q9LXY6, Q9LYC1, Q9M099, Q9M7I7, Q9M9Q6, Q9MA46, Q9MAA7, Q9MAR8, Q9S745, Q9SFB5, Q9SG92, Q9SIN9, Q9SJI7, Q9SMM9, Q9SMN0, Q9SQR3, Q9SQX6, Q9SR22, Q9SR23, Q9SU71, Q9SU72, Q9SV02, Q9SV04, Q9SX25, Q9SX78, Q9SZU7, Q9XF23, Q9ZQ91, Q9ZQQ0, Q9ZUG3, Q9ZUN1, Q9ZVN2, RPH51899, WP_113953187, WP_118956249, WP_118981493, WP_135574293, WP_135684980, WP_135778549, WP_147167049, WP_68335717, WP_84122819, WP_84406699, WP_98075253, XP_12750889, XP_1701466, XP_17603429, XP_18838334, XP_20435393, XP_21678655, XP_2298118, XP_26193674, XP_30950868, XP_34901907, XP_3610038, XP_5538874, XP_5705377, XP_5712425, XP_5714234, XP_5714441, XP_5717076, XP_644406,

**Benefits and limitations of extracting KEGG metabolic pathways**

We identified gene candidates involved in starch and sucrose metabolism by comparing their recovery against the established KEGG metabolic pathway (Fig S5, Table S3). The benefit of this graphic method is that users can get a broad overview and then extract genes present in *C. tuberculosum* (Fig S5, Table S3, Table S4). At a glance, several genes in this pathway are involved in the production of glucose-based polymers, likely including Floridean starch, and can be identified for further sequence characterization or experimental follow up. However, KEGG annotations are based on a limited number of datasets composed predominantly from model-organism. Therefore, the utility of KEGG annotations for gene discovery in non-model organisms such as *C. tuberculosum* is limited. Algal specific genes not present in the starch and sucrose metabolic pathways would require manual identification and verification. This could be achieved by creating algal gene specific hidden Markov models constructed based on alignment of conserved sequences or search using BLAST against the transcriptome. For this reason, the methods described here are best utilized as a first pass screening to identify sequences of interest from established a priori metabolic pathways.

**References**

1. Parra G, Bradnam K, Korf I. CEGMA: A pipeline to accurately annotate core genes in eukaryotic genomes. Bioinformatics. 2007;23: 1061–1067. doi:10.1093/bioinformatics/btm071

2. Simão FA, Waterhouse RM, Ioannidis P, Kriventseva E V., Zdobnov EM. BUSCO: Assessing genome assembly and annotation completeness with single-copy orthologs. Bioinformatics. 2015;31: 3210–3212. doi:10.1093/bioinformatics/btv351

3. Brawley SH, Blouin NA, Ficko-Blean E, Wheeler GL, Lohr M, Goodson H V., et al. Insights into the red algae and eukaryotic evolution from the genome of Porphyra umbilicalis (Bangiophyceae, Rhodophyta). Proceedings of the National Academy of Sciences of the United States of America. 2017;114: E6361–E6370. doi:10.1073/pnas.1703088114

4. Boerjan W, Ralph J, Baucher M. Lignin Biosynthesis. Annual Review of Plant Biology. 2003;54: 519–546. doi:10.1146/annurev.arplant.54.031902.134938

5. Vogt T. Phenylpropanoid Biosynthesis. Molecular Plant. 2010;3: 2–20. doi:https://doi.org/10.1093/mp/ssp106

6. Hamberger B, Bak S. Plant P450s as versatile drivers for evolution of species-specific chemical diversity. Philosophical transactions of the Royal Society of London Series B, Biological sciences. 2013;368: 20120426. doi:10.1098/rstb.2012.0426

7. Quinlan RF, Shumskaya M, Bradbury LMT, Beltrán J, Ma C, Kennelly EJ, et al. Synergistic Interactions between Carotene Ring Hydroxylases Drive Lutein Formation in Plant Carotenoid Biosynthesis. Plant Physiology. 2012;160: 204 LP – 214. doi:10.1104/pp.112.198556

8. Hasemann CA, Kurumbail RG, Boddupalli SS, Peterson JA, Deisenhofer J. Structure and function of cytochromes P450: a comparative analysis of three crystal structures. Structure (London, England : 1993). 1995;3: 41–62. doi:10.1016/s0969-2126(01)00134-4

9. Bak S, Beisson F, Bishop G, Hamberger B, Höfer R, Paquette S, et al. Cytochromes p450. The arabidopsis book. 2011/10/06. 2011;9: e0144–e0144. doi:10.1199/tab.0144

10. Ye ZH, Kneusel RE, Matern U, Varner JE. An alternative methylation pathway in lignin biosynthesis in Zinnia. The Plant Cell. 1994;6: 1427 LP – 1439. doi:10.1105/tpc.6.10.1427

11. Ferrer J-L, Zubieta C, Dixon RA, Noel JP. Crystal Structures of Alfalfa Caffeoyl Coenzyme A 3-O-Methyltransferase. Plant Physiology. 2005;137: 1009 LP – 1017. doi:10.1104/pp.104.048751

12. Walker AM, Sattler SA, Regner M, Jones JP, Ralph J, Vermerris W, et al. The Structure and Catalytic Mechanism of Sorghum bicolor Caffeoyl-CoA O-Methyltransferase. Plant physiology. 2016/07/25. 2016;172: 78–92. doi:10.1104/pp.16.00845

13. Lam KC, Ibrahim RK, Behdad B, Dayanandan S. Structure, function, and evolution of plant O-methyltransferases. Genome. 2007;50: 1001–1013. doi:10.1139/G07-077

14. Vanholme R, Cesarino I, Rataj K, Xiao Y, Sundin L, Goeminne G, et al. Caffeoyl Shikimate Esterase (CSE) Is an Enzyme in the Lignin Biosynthetic Pathway in Arabidopsis. Science. 2013;341: 1103 LP – 1106. doi:10.1126/science.1241602

15. Saleme M de LS, Cesarino I, Vargas L, Kim H, Vanholme R, Goeminne G, et al. Silencing Caffeoyl Shikimate Esterase Affects Lignification and Improves Saccharification in Poplar. Plant Physiology. 2017;175: 1040 LP – 1057. doi:10.1104/pp.17.00920
