# Supplementary figures and images for: Transcriptome of the coralline alga Calliarthron tuberculosum (Corallinales, Rhodophyta) reveals convergent evolution of a partial lignin biosynthesis pathway
Source: PLoS One. 2022 Jul 14;17(7):e0266892. doi: 10.1371/journal.pone.0266892 (PMC9282553; doi:10.1371/journal.pone.0266892)

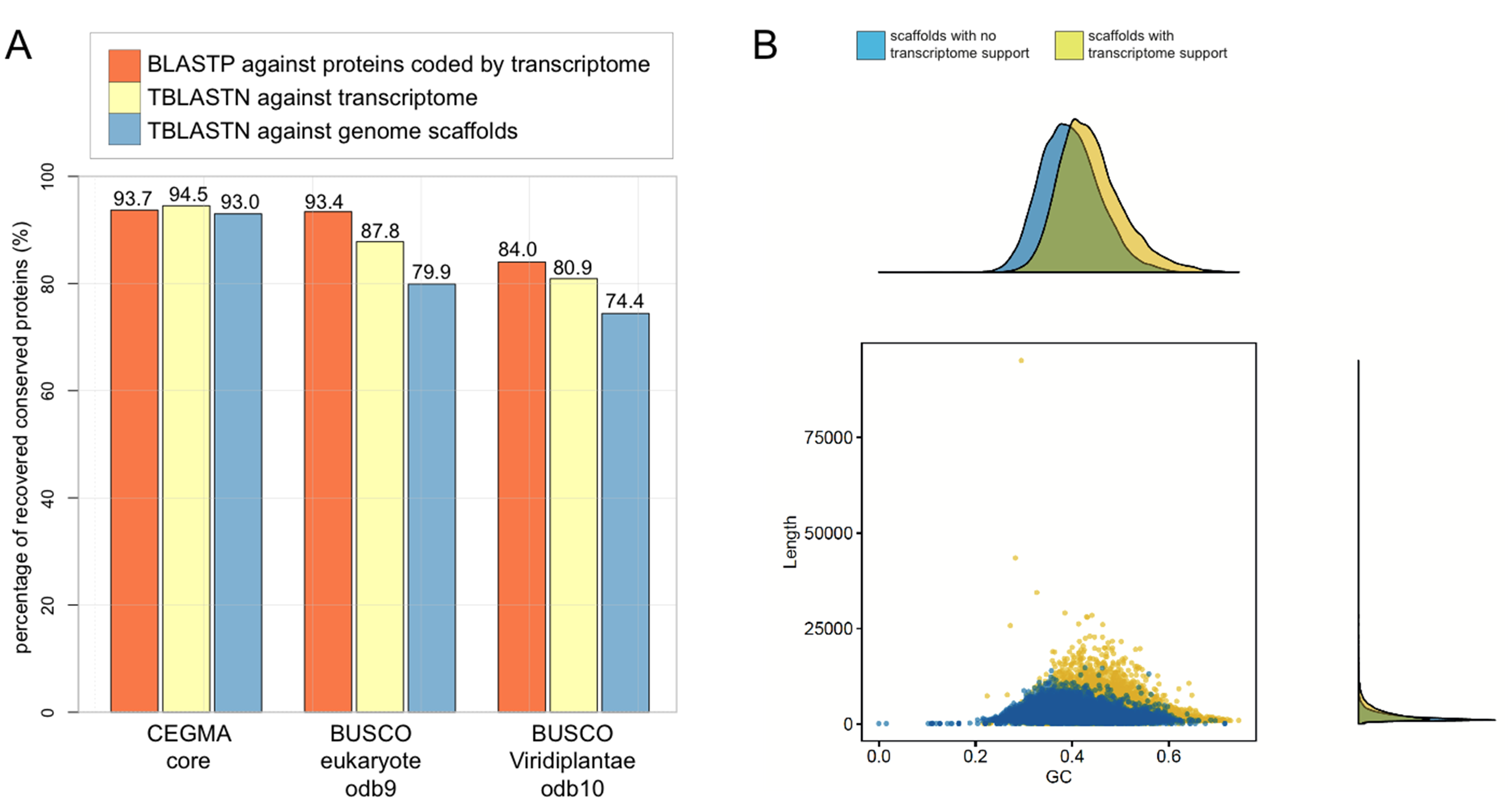

Supplement: S1 Fig — (A) Transcriptome sequences show high recovery of eukaryotic genes in CEGMA/BUSCO analysis. Percentage of genomic scaffolds with transcriptome support and transcriptomic scaffolds alone that share amino acid sequences with the core eukaryotic gene databases including CEGMA, BUSCO eukaryotic, and BUSCO Viridiplantae. Transcriptome encoded amino acid sequences were searched against the databases using BLASTP (orange) or TBLASTN (yellow), and genomic scaffolds were searched against the databases using TBLASTN (blue). (B) Transcriptomic support of genomic data analyzed by GC content and transcript length. The distribution of GC content (above) against transcript lengths is shown for scaffolds with transcriptome support (blue) and scaffolds without transcriptome support (yellow) (right). (TIF) [file pone.0266892.s002.tif]

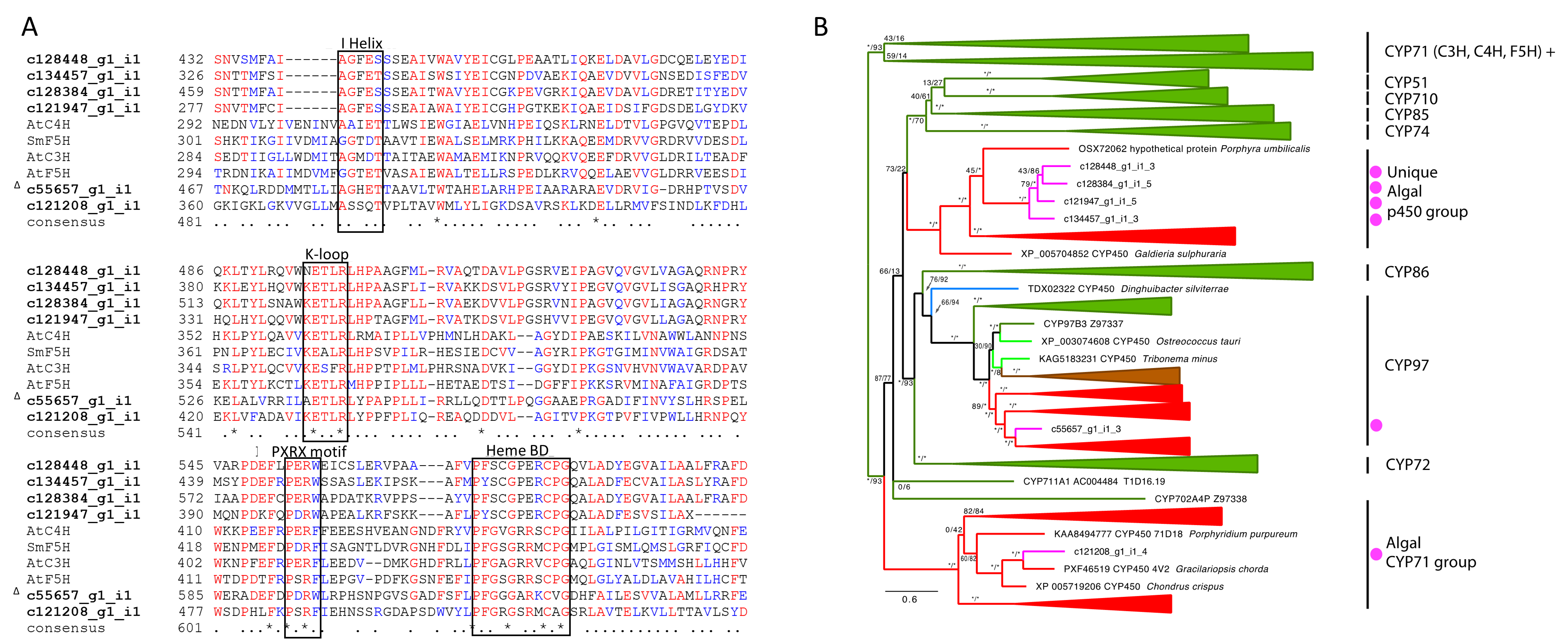

Supplement: S2 Fig — (A) Partial alignment of C. tuberculosum P450 candidates with C3H, C4H, and F5H from A. thaliana, and a novel F5H from Selaginella moellendorffii. Heme binding domain residues, secondary structure stabilizing K helix residues, PXRX, and the I-helix are indicated [8]. Sites with <80% coverage were removed. A strong candidate for beta-carotene synthesis is indicated with a triangle. (B) Unrooted CYP450 maximum likelihood gene tree with C. tuberculosum (magenta dots) and additional taxa (Embryophyta–dark green, Chlorophyta–light green, Rhodophyta–red, Animalia and Opisthokonta–purple, Bacteria and Cyanobacteria–blue, Oomycota, Mycetozoa and Fungi–yellow, Ochrophyta–brown). Functionally demonstrated plant C3H, C4H, and F5H are labeled (+). Additional functional groups are labeled [9]. Ultrafastbootstrap values > 95 are marked by *. Model = VT+F+G4. (TIF) [file pone.0266892.s003.tif]

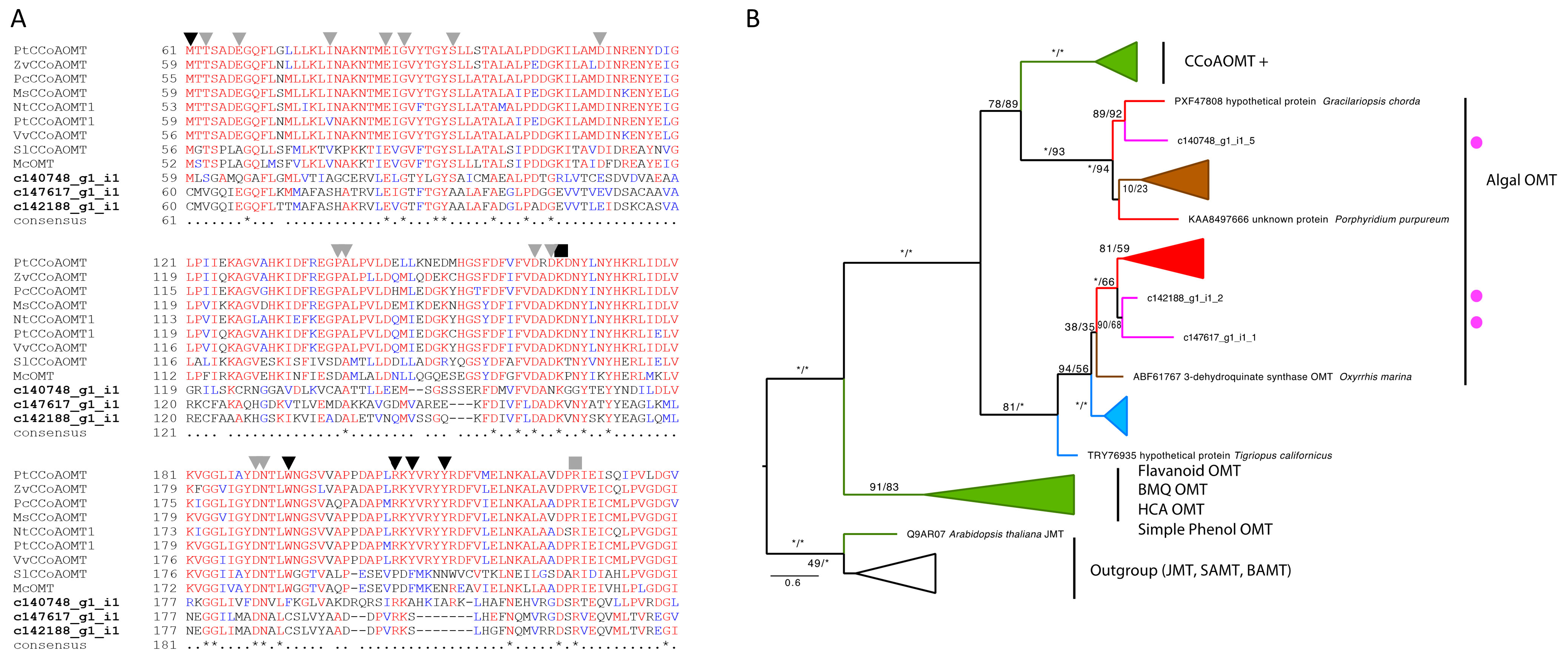

Supplement: S3 Fig — (A) Partial alignment of C. tuberculosum CCoAOMT sequence candidates with CCoAOMT from land plants. Substrate recognition residues (black triangle), divalent metal ion and cofactor binding residues (grey triangle), catalytic residues (back square), and the positively charged R220 necessary for substrate recognition (grey square) are indicated. Sites with < 70% coverage were removed. (B) Unrooted maximum likelihood gene tree of biochemically characterized plant O-methyltransferases with C. tuberculosum (magenta dots) and additional taxa (Embryophyta–dark green, Chlorophyta–light green, Rhodophyta–red, Animalia and Opisthokonta–purple, Bacteria and Cyanobacteria–blue, Oomycota, Mycetozoa and Fungi–yellow, Ochrophyta–brown). Functionally demonstrated plant CCoAOMT are labeled (+). Additional functional groups are labeled [13]. Ultrafastbootstrap values > 95 are marked by *. Model = LG + G4. JMT, SAMT, and BAMT are closely related to OMTs. (TIF) [file pone.0266892.s004.tif]

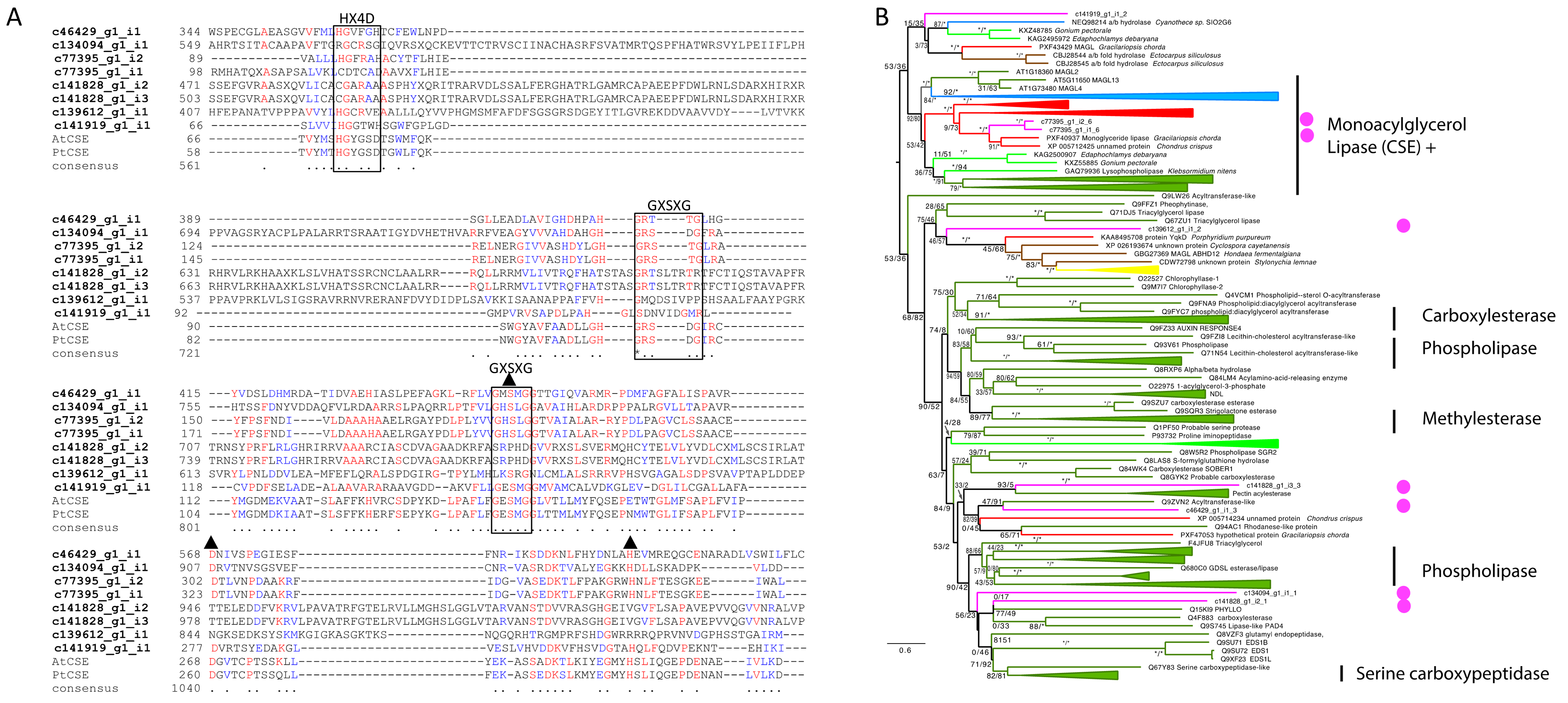

Supplement: S4 Fig — (A) Partial alignment of C. tuberculosum CSE sequence candidates with CSE from land plants. Acyl transferase motifs (HX4D), lipase motifs (GXSXG) and active site residues (triangle) are indicated. Sites with < 70% coverage were removed. (B) Unrooted maximum likelihood gene tree of C. tuberculosum CSE candidates (magenta dots) and additional taxa (Embryophyta–dark green, Chlorophyta–light green, Rhodophyta–red, Animalia and Opisthokonta–purple, Bacteria and Cyanobacteria–blue, Oomycota, Mycetozoa and Fungi–yellow, Ochrophyta–brown). Functionally demonstrated plant CSE are labeled (+). Additional functional groups are labeled. Ultrafastbootstrap values > 95 are marked by *. Model = VT+G4. (TIF) [file pone.0266892.s005.tif]

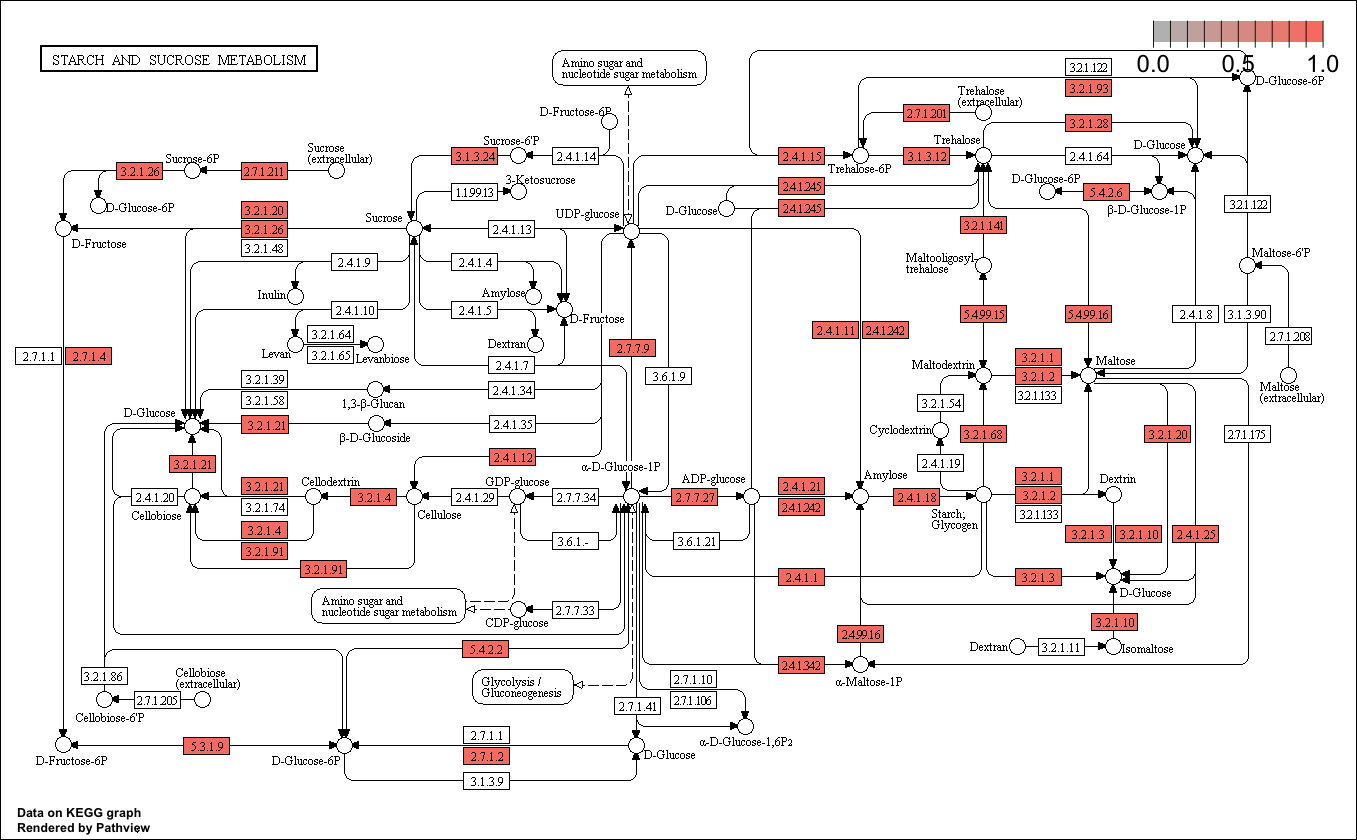

Supplement: S5 Fig — KEGG based annotation showing the starch and sucrose metabolic pathway with C. tuberculosum annotations highlighted. The gradient map in the top right corner indicates the level of transcription, with white and dark pink coloring representing absence and presence of expression respectively. The annotated map, number “00500”, was extracted in the provided R file using the pathview program. (TIF) [file pone.0266892.s006.tif]
